# Invasion intensity influences scale-dependent effects of an exotic species on native plant diversity

**DOI:** 10.1038/s41598-019-55165-z

**Published:** 2019-12-10

**Authors:** Thomas J. Valone, David P. Weyers

**Affiliations:** Department of Biology, Saint Louis University, St. Louis, MO USA

**Keywords:** Biodiversity, Community ecology, Conservation biology

## Abstract

Invasive plant species reduce the diversity of natives by altering habitats or disturbance regimes, but it is less clear whether they do so via competitive exclusion. Here, we show that invader abundance alters scale-dependent competitive effects of invasion on native plant richness. Large-seeded exotic annual *Erodium cicutarium* invaded a site that manipulated rodent granivores. The invader became dominant on all plots but attained its highest abundance on plots that removed rodents. Invasion reduced plant abundance but not evenness; site-wide richness did not change over time on control plots but declined significantly on rodent removal plots. Species-area relationships within plots changed differently with invasion intensity: slopes increased and y-intercepts decreased on control plots relative to rodent removal plots. Changes in species-area slopes and y-intercepts following invasion suggest that common rather than rare species were most strongly impacted at small spatial scales on control plots, while common and rare species were both negatively impacted at all spatial scales on rodent removal plots. Small-seeded species declined in abundance following invasion more so than large-seeded species, indicative of competitive interactions mediated by seed size. These results reveal variation in scale-dependent competitive effects of invasion on native richness associated with invasion intensity.

## Introduction

Human activities have led to the establishment of numerous exotic species. Invasive exotics attain high abundance and negatively impact natives often by altering disturbance regimes and habitat structure, introducing novel diseases, and by consuming them^1–6^.

Less clear is whether invasive plant species pose a global threat to biodiversity^7–11^, because evidence of extinctions caused solely by invasive plants is rare^12–16^. Yet, comparisons of uninvaded and heavily invaded sites often show reduced native richness in the latter^17–19^ and negative correlations between the abundance of an invasive species and native species richness are common^20^. However, invasive plants can also enhance species diversity through facilitation^21–23^. In addition, in many locations, diversity has increased because exotic species additions have exceeded extirpations^11,24–26^.

One explanation for the above invasion paradox^27^ involves spatial scale. Studies conducted at broad spatial scales often observe positive associations between exotic species and native richness while those focusing on smaller scales (<25 m^2^) often report negative relationships^22,27,28^. However, recent analyses of work at small scales have found a lack of consistent relationships between native and exotic richness^29,30^ and the impact of a single invasive plant on native richness can range from positive, to neutral, to negative^31^. In addition, analyses of time series show no consistent negative trends in community richness for invaded communities at small spatial scales^32–35^. It has also been difficult to predict how invasion might affect particular native species. For instance, it is unclear whether invasions will more often negatively impact common or rare species in communities^6,36^.

**Figure 1:**
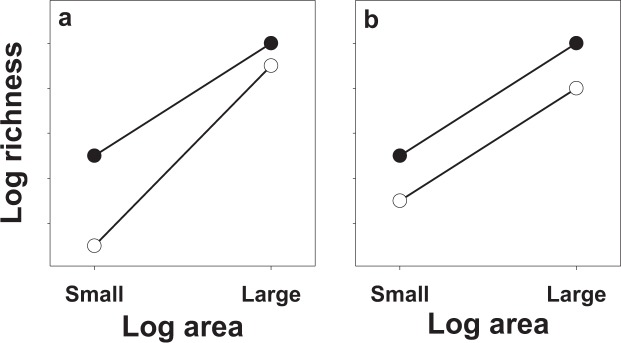
Two possible outcomes of the negative effects of exotic species invasion on native plant species richness for a community with low evenness. (**a**) When common species are more negatively impacted than rare species, there is a greater decrease in species richness at small than large spatial scales. (**b**) When common and rare species are equally negatively impacted, there is a similar decrease in richness at the small and large spatial scales.

Recent theoretical work has sought to clarify these issues by focusing on how invasions influence species-area relationships (SAR)^10,33,37–39^. Using spatially explicit models, Powell *et al*.^28^ examined how invasion by an invasive species impacted native species richness in scenarios that differed in competitive impacts of the invader on common and rare species. For communities with typically low evenness (i.e., communities that contain few common but many rare species), when the invader most strongly impacted common species (perhaps by sharing important niche characteristics with them), the invaded community exhibited a steeper SAR slope and strong reduction in the y-intercept compared to the pre-invaded community, indicating that invasion had strong effects on species richness at small scales but weaker negative impacts at larger scales (Fig. 1a). However, when the invader negatively affected common and rare species equally (perhaps by competitively dominating both), there was no change in the slope of the SAR following invasion and weaker reductions in the y-intercept, a pattern suggesting similar negative effects of invasion on native richness across scales (Fig. 1b). The competitive effects of invasion are often density-dependent^40–44^ with stronger negative effects on natives as invader density increases. As such, Rejmánek & Stohlgren^45^ suggest that the competitive effects of an invasive species on SAR may be influenced by invasion intensity, with the first pattern (Fig. 1a) most likely at modest invader densities and the second (Fig. 1b) associated with higher invader abundances, but this prediction lacks empirical evaluation.

Previous analyses of scale-dependent impacts of invasion have compared invaded versus non-invaded sites^46,47^. Such comparisons involve assumptions about the comparability of sites that may have differed markedly before invasion and so need to be interpreted cautiously^45,48–50^. An approach that ameliorates these concerns would examine the same sites before and after invasion^49^, in order to better determine how invasion *per se* impacted native species diversity, as well as common versus rare species, but such data are rare^48^.

Here, we examine a time series of data from multiple plots at a single site that span a dramatic increase in the abundance of a large-seeded exotic invasive annual plant, *Erodium cicutarium*. In the pre-invasion time period examined (1989–1995), native species dominated all plots. In the post-invasion time period (1998–2005), the invader dominated all plots but was significantly more abundant on plots that had removed rodents compared to control plots. These data provide an opportunity to compare SAR changes following invasion on control versus rodent removal plots to test how invasion intensity influences species-area relationships and impacts on common versus rare species. We predict that SAR slopes will increase and y-intercepts will decline more strongly on control than rodent removal plots following invasion, a pattern consistent with the predicted effects of invasion intensity outlined by Rejmánek & Stohlgren^45^. Next, we examine each species’ invasion response, its change in relative abundance following invasion, to determine how common and rare species were impacted. In plant communities, the relative abundance of a species in a community is often related to its seed mass: small-seeded species can and often do obtain high abundance while large-seeded species rarely do so^51^. Seed size is also thought to affect interspecific competitive ability: large-seeded species often outcompete small-seeded species for resources such as light and water and are more tolerant of environmental stress^52–54^. If *E. cicutarium* outcompeted native species, we predict that small-seeded, common species should be more negatively impacted by invasion than large-seeded, rare species.

## Results

In the pre-invasion time period, native *Machaerantha gracilis* was dominant, averaging over 40% of all individuals recorded on all plots, while *E. cicutarium* was rare, averaging less than 5% of individuals (Supplementary Tables S1, S2). In the post-invasion time period, *E. cicutarium* dominated all plots, averaging 53% and 69% of individuals per year, on control and rodent removal plots respectively, while *M. gracilis* became rare, averaging <1% of individuals recorded on all plots (Tables 1, S1, S2).

**Table 1:** Mean (s.e.m.) abundance per plot (number of individuals), site-wide richness, plot evenness, and fractional abundance of the invader by treatment (C: control; R: rodent removal) in each time period and the results of statistical tests across time periods.

Parameter	Treatment	Time Period		t	P-value
		Pre-invasion	Post invasion		
Abundance	C	2410.4 (232.1)	627.9 (60.6)	8.23	<0.001
	R	2714.4 (398.1)	963.3 (112.1)	5.42	0.004
Richness	C	27.8 (0.7)	28.2 (1.2)	−0.35	0.74
	R	28.7 (1.1)	21.8 (1.5)	5.25	0.003
Evenness	C	0.52 (0.02)	0.53 (0.03)	−0.45	0.66
	R	0.49 (0.02)	0.40 (0.05)	2.15	0.08
Fractional abundance of *E. cicutarium*	C	0.01 (0.01)	0.53 (0.04)	−11.94	<0.001
	R	0.04 (0.01)	0.69 (0.03)	−21.0*	<0.001

*Wilcoxon W test statistic.

On both types of plots, mean community abundance was significantly lower in the post-invasion period, but community evenness did not differ across time periods (Table 1). On control plots, mean yearly site-wide richness did not differ across time periods; it declined significantly for the rodent removal plots in the post-invasion period (Table 1).

Invasion affected SAR slopes and intercepts (Supplementary Fig. S1) as predicted. On control plots, Δ slope (post-invasion – pre-invasion) increased and Δ y-intercept decreased on 9 of 10 plots. In contrast, Δ slope increased on just half of the rodent removal plots, while Δ y-intercepts declined on four of the six plots. Mean Δ slope was significantly higher on control plots compared to rodent removal plots (control mean [s.e.m.] Δ slope = 0.09 [0.02]; rodent removal mean [s.e.m.] Δ slope = −0.02 [0.04], t = 2.56, P = 0.011, one-tailed t-test). In addition, Δ y-intercept was significantly lower on control compared to rodent removal plots (control mean [s.e.m.] Δ y-intercept = −0.57 [0.10]; rodent removal mean [s.e.m.] Δ y-intercept = −0.21 [0.18], t = 1.89, P = 0.04, one-tailed t-test).

**Figure 2:**
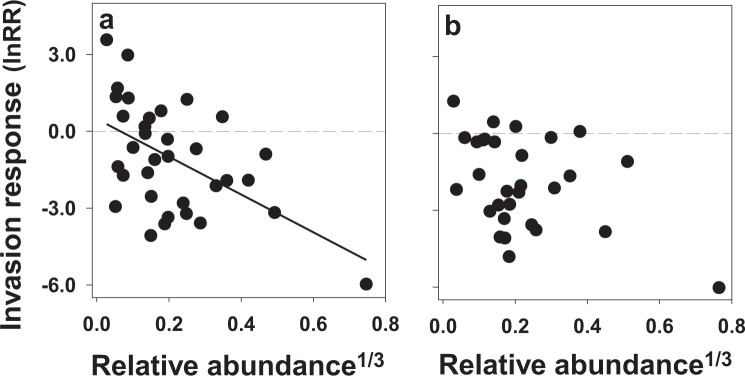
Relationships between invasion response (lnRR) and the cube-root of pre-invasion relative abundance for each species in (**a**) control, (**b**) rodent removal plots. Negative values represent declines in abundance after invasion. A simple linear regression was used to evaluate relationships.

**Figure 3:**
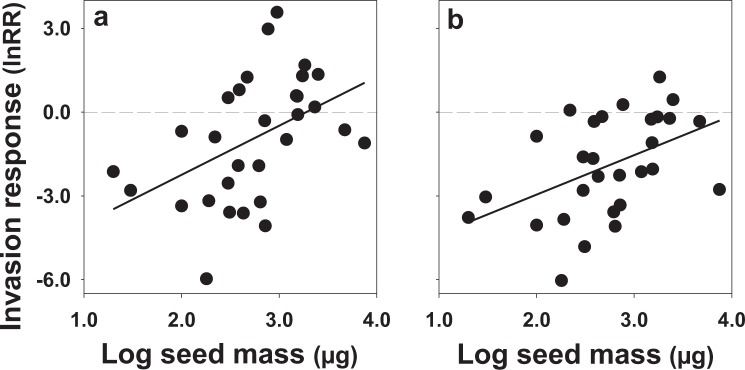
Log response ratio (lnRR) of species for (**a**) control, (**b**) rodent removal treatments as a function of seed mass. A simple linear regression was used to evaluate relationships.

For control plots, common species were more negatively impacted by invasion than rare species (i.e., there was a significant negative relationship between invasion response and pre-invasion relative abundance) (y = –7.4x + 0.49, t = −3.60, P = 0.001, N = 33) (Fig. 2a), a relationship robust to removal of the most common species from the analysis (y = −6.3x + 0.31, t = −2.4, P = 0.02, N = 32). On rodent removal plots, the relationship was less clear: using all species, the relationship was negative (y = –4.76x − 0.88, t = −2.37, P = 0.025, N = 30), but the relationship was not significant after removal of the most common species (y = −2.5x − 0.92, t = −0.92, P = 0.37, N = 29) (Fig. 2b) indicating that both common and rare species were negatively impacted by invasion (i.e., they exhibited similar, negative invasion responses). Finally, there was a significant positive relationship between invasion response and seed mass on both control and rodent removal plots suggesting that invasion negatively impacted small seeded-species more so than large-seeded species (control plots: y = 1.8x − 5.7, t = 2.8, P = 0.01; rodent removal plots: y = 1.4x − 5.9, t = 2.7, P = 0.012; Fig. 3).

## Discussion

Our analyses are unique in that we examined the same communities before and after invasion by a dominant exotic species. On all plots, invasive *E. cicutarium* replaced a native as the numerically dominant species in the post-invasion time period. We found that the impacts of invasion on native community richness were scale-dependent and differed considerably with invasion intensity. While we observed strong reductions in native richness at the smallest spatial scale on all plots, there were different impacts on richness at the larger scales examined. On control plots, there was no difference in site-wide yearly mean richness across time periods (Table 1), but invasion did result in modest reductions in richness at the largest scale within plots (4.0 m^2^ sampled area), and stronger reductions in richness at the smallest spatial scales (Supplementary Fig. S1). In contrast, on rodent removal plots, we observed a significant reduction in site-wide yearly mean richness (Table 1) as well as strong reductions in native richness at all scales measured within plots (Supplementary Fig. S1).

The contrasting SAR patterns from the control and rodent removal plots provide the first empirical evidence that invader abundance can alter the shape of the SAR as suggested by Rejmánek & Stohlgren^45^. The increase in SAR slope on control plots post-invasion (i.e., higher Δ slope and lower Δ y-intercept compared to rodent removal plots) (Supplemental Fig. S1) parallel’s previous work^28,46^ and suggests that invasion may often strongly impact common, rather than rare species (but see^47^). In contrast, richness declined similarly at all spatial scales within rodent removal plots leading to smaller Δ slope and Δ y-intercept values on these plots compared to controls (Supplementary Fig. S1). We suggest that post-invasion SAR patterns differ across plot types because invasion intensity (abundance of *E. cicutarium)* was significantly higher on plots that had removed rodent granivores (Table 1).

Three lines of evidence suggest that a competitive interaction between *E. cicutarium* and natives, mediated by differences in seed size, provides a mechanism generating the above patterns. First, prior to invasion, the most common species in the annual community produced seeds of small size (Supplementary Tables 1, 2), a pattern found in many plant communities^51,55^. Such species might be most vulnerable to invasion by a competitively dominant exotic that produces relatively large seed such as *E. cicutarium* because seed mass often correlates with competitive ability^52,54^. In contrast, large-seeded species were relatively rare prior to invasion (Supplementary Tables 1, 2) and were not as negatively impacted as small-seeded species by invasion, especially on control plots (Fig. 3). Second, *E. cicutarium* tends to germinate early in the winter season^56^, and is a rosette-forming annual^57^, which can give it a competitive advantage over native species that germinate later in the growing season. And, third, experimental removal of *E*. *cicutarium* in this system leads to higher abundance and diversity of natives^58,59^, characteristic of a competitive interaction.

Few studies have examined how exotic invasion affects SAR. Powell *et al*.^46^ compared the SAR of invaded versus non-invaded plant communities in three disparate systems in North America. At each site, the invaded community had a greater slope and lower y-intercept than the non-invaded community because invasion reduced richness at the small scales to a greater degree than at the large scales. Stohlgren & Rejmánik^60^ examined SAR from hundreds of plant communities that differed in invasion intensity. They reported much variation in SAR slopes and intercepts across sites but their data were skewed toward sites dominated by natives: over 70% of sites had <5% exotic cover, while less than 5% of the sites had >50% exotic cover^49^. Neither study, however, could provide a mechanistic explanation for the patterns observed. We show that invasion most strongly impacted native richness at the smallest spatial scale observed in a manner consistent with competitive dominance because small-seeded common species were more negatively impacted by invasion than large-seeded rare species. Competitive interactions in plant communities are known to be scale-dependent and strongest at small scales^61^.

In this and other systems, invasion most negatively impacted common rather than rare species, especially on control plots. If a general result, this pattern has implications for how plant invasions may impact ecosystem function. Dominant species are often important providers of ecosystem services; a reduction in their abundance can be just as important as a reduction in richness in reducing ecosystem function^32,62,63^. However, dominant invaders can also provide ecosystem services, in lieu of common natives^64–66^. Thus, invasions that reduce common natives and reduce diversity may not necessarily result in a reduction in ecosystem services. Our findings suggest that further research is needed to characterize invasion impacts on common and rare species and resulting impacts on communities and ecosystem services^67^.

## Conclusions

In summary, invasion by exotic *E. cicutarium* negatively impacted the richness of native species in this system. However, the severity of such impacts varied with spatial scale and invader abundance. At modest invasion intensity, *E. cicutarium* most negatively impacted small-seeded, common rather than large-seeded, rare species, most likely due to a competitive interaction mediated by seed size. However, at higher invasion intensity, the invader negatively impacted both common and rare species at all spatial scales. Our work helps to clarify the invasion paradox because it shows how both spatial scale and invasion intensity impact native richness following invasion by a dominant exotic species.

## Methods

### Site description

Data come from a 20 ha Chihuahuan desert scrubland site established in 1977 near Portal, Arizona, U.S.A.^68^. Dominant perennial vegetation includes *Acacia, Prosopis, Ephedra*, and *Flourensia* shrubs along with scattered perennial grasses^69^. The granivorous rodent community at the site is diverse and includes kangaroo rats in the genus *Dipodomys* and mice, mainly in the genera *Chaetodipus*, *Perognathus*, and *Reithrodontomys*. These species feed predominantly on the seeds of winter annual plants^70^.

The site contains 24, 0.25 ha plots that manipulate rodent granivory. Here we focus on six plots that exclude all rodents (rodent removal plots) and 10 plots that allow access to all rodents (control plots)^68^. Rodents have been continuously censused on all plots each month since 1977 and census data indicate that the rodent removal treatment is highly effective at minimizing the abundance of rodents on these plots^71^.

Winter annual plant communities in southwestern North America germinate in response to seasonal precipitation that typically falls between December and March. Since 1989, we have counted the abundance of all annual plants rooted within 16 0.25 m^2^ fixed sampling locations within each plot. Annual plant censuses occur in late March or early April each year, at the end of the growing season when most species are either flowering or setting seed. About 40 species of winter annual plants have been recorded at the site, although the number observed each year ranges widely because the abundance and diversity of these communities varies with the timing and amount of winter precipitation^72^. The seed size of the winter annual species at the site ranges from 0.002 to over 7.0 mg^73^.

Exotic annual *Erodium cicutarium* (seed mass: 0.99 mg) has been present at the site since 1977. Prior to 1995, *E. cicutarium* was rare, typically representing <5% of the annual plants counted in a given year, although granivorous rodent removal treatments resulted in increased *E. cicutarium* abundance, suggesting that rodent granivory regulates its abundance^70^. The exotic increased in abundance dramatically at the Portal site in the mid-1990s, during a three-year period when rodent abundance was low^74^. By the late 1990s, over 40% of the individual winter annual plants counted on plots each year were *E. cicutarium* and this large-seeded exotic dominated the winter annual plant community thereafter: it had become an invasive species at the site^59^.

### Time periods

Because the annual plant community varies each year with seasonal precipitation, we examined two time periods around the large increase in abundance of *E. cicutarium*. Examination of multiple years within each time period allows a more representative characterization of the plant communities on plots before and after the invader became abundant. The pre-invasion time period was defined as 1989–1995 while the post-invasion time period was 1998–2005. However, 1990, 1999 and 2000 were years of little winter precipitation and so no winter annual plant germination was observed, leaving six years of data for analysis in each time period.

### Community parameters

We first examined how invasion by *E. cicutarium* affected the native plant community by comparing mean community abundance (number of individuals per plot), richness, and evenness on plots in each time period. For evenness, we calculated each species’ yearly fractional abundance for each plot. We used these values to calculate Pielou’s J^75^ each year in each plot as our measure of plot evenness using each year’s plot richness in the denominator (J = H’/H’_max_ = [−Σ(p_i_)ln(p_i_)]/ln S) where H’ is the Shannon-Wiener index of species diversity, p_i_ is the proportion of total abundance represented by the i^th^ species, and S is the number of species in the community. We tested for differences across time periods in mean plot community abundance, richness, and evenness using two-tailed, paired t-tests, after testing for normality, using N = 10 control, or N = 6 rodent removal plots. For non-normal data, we used a Wilcoxon signed-rank test.

### SAR slopes and intercepts

We conducted species-area regressions within each plot, each year for both time periods. For each plot each year, we began with a single 0.25 m^2^ area quadrat in a randomly selected corner and determined the number of native species recorded (Supplementary Fig. S2). Next, we increased spatial scale by examining the four nearest corner quadrats (1.0 m^2^ area sampled) to determine species richness at this scale. We increased spatial scale again by next examining the nine nearest corner quadrats (2.25 m^2^ area sampled), and finally, we calculated richness using all 16 quadrats (4.0 m^2^ area sampled) (Supplementary Fig. S2). This procedure yielded a single SAR slope and y-intercept for each plot each year. We then averaged these values over each time period for each plot to obtain a single SAR slope and y-intercept for each plot for each time period. To test the predicted changes in SAR slopes and y-intercepts following invasion across plot types, we calculated Δ slope and Δ y-intercept, the change in SAR slope and y-intercept, on each plot following invasion (post invasion value – pre-invasion value), and tested for differences using a t-test with N = 10 control and N = 6 rodent removal plots.

### Invasion response

To examine the impact of invasion on common versus rare species, we calculated each species’ invasion response as the mean log response ratio, lnRR, using the mean plot abundance of a species within each treatment over each time period, where lnRR = (ln[post-invasion mean plot abundance]/[pre-invasion mean plot abundance]). To do this, we combined the abundance data for all species over all control plots or over all rodent removal plots each year within each time period to calculate each species’ mean relative abundance in each time period on the different types of plots. We then plotted each species’ invasion response as a function of its pre-invasion relative abundance (cube root transformed to improve homoscedasticity) and conducted a simple linear regression, after testing for normality.

We also plotted each species’ invasion response as a function of its seed mass log_10_ (μg) and conducted a simple linear regression, after testing for normality.

### Seed mass data

Seed mass data were obtained from Chen and Valone^73,76^ and supplemented with Kew Royal Botanical Gardens Seed Information Database available from www.kew.org.

## Supplementary information

Supplemental Information

## Data Availability

Data used for analyses are available at https://doi.org/10.5281/zenodo.1215988.
