# Supplementary material for: Invasion intensity influences scale-dependent effects of an exotic species on native plant diversity
Source: Sci Rep. 2019 Dec 10;9:18769. doi: 10.1038/s41598-019-55165-z (PMC6904574; doi:10.1038/s41598-019-55165-z)
Supplement: Supplementary file 1 — Supplemental Information [file 41598_2019_55165_MOESM1_ESM.pdf]

Invasion intensity influences scale-dependent effects of an exotic species on native plant diversity

Supplementary Information

Thomas J. Valone & David Weyers

Table S1: Winter annual plant species, mean (s.e.m.) fractional abundances observed in both pre-invasion and post-invasion time periods ranked from most to least abundant in the control plots, seed mass, and family.

Pre-invasion rank abundance	Species	Pre-invasion fractional abundance	Post-invasion fractional abundance	Seed mass (mg)	Family
1	*Machaeranthera (=Haplopappus) gracilis*	0.416(0.119)	0.005(0.002)	0.18	Asteraceae
2	*Eriastrum diffusum*	0.120(0.026)	0.013(0.005)	0.19	Polemoniaceae
3	*Eriogonum abertianum*	0.103(0.027)	0.113(0.044)	0.22	Polygonaceae
4	*Vulpia octoflora*	0.074(0.035)	0.024(0.006)	0.38	Poaceae
5	*Spermolepis echinata*	0.047(0.013)	0.018(0.011)	0.62	Apiaceae
6	*Astragalus nuttallianus*	0.042(0.038)	0.041(0.017)	1.53	Fabaceae
7	*Cryptantha micrantha*	0.036(0.015)	0.012(0.008)	0.02	Boraginaceae
8	*Gilia sinuata*	0.024(0.008)	0.003(0.002)	0.31	Polemoniaceae
9	*Descurainia pinnata*	0.021(0.006)	0.027(0.006)	0.1	Brassicaceae
10	*Chaenactis stevioides*	0.016(0.013)	0.090(0.020)	0.47	Asteraceae
11	*Stephanomeria exigua*	0.015(0.006)	0.001(0.0004)	0.64	Asteraceae
12	*Erigeron divergens*	0.014(0.009)	0.002(0.002)	0.03	Asteraceae
13	*Erodium cicutarium*†	0.012(0.005)	0.534(0.081)	0.99	Geraniaceae
14	*Malacothrix fendleri*	0.008(0.006)	0.0003(0.0002)	0.1	Asteraceae
15	*Plantago patagonica (=purshii)*	0.008(0.002)	0.008(0.002)	1.19	Plantaginaceae
16	*Phacelia arizonica (=popei)*	0.008(0.004)	0.008(0.007)	0.71	Boraginaceae
17	*Baileya multiradiata*	0.007(0.005)	0.001(0.0004)	0.43	Asteraceae
18	*Chenopodium fremontii*	0.006(0.003)	0.009(0.008)	0.39	Chenopodiaceae
19	*Lupinus concinnus*	0.004(0.002)	0.005(0.003)	7.5	Fabaceae
20	*Lepidium lasiocarpum*	0.003(0.002)	0.001(0.0004)	0.3	Brassicaceae
21	*Calycoseris wrightii*	0.003(0.002)	0.00003(0.0003)	0.72	Asteraceae
22	*Cryptantha crassisepala*	0.003(0.001)	0.006(0.002)	0.3	Boraginaceae
23	*Lupinus sp. 2*	0.003(0.003)	0.0005(0.0003)	NA††	Fabaceae
24	*Microseris lindleyi (=Uropappus lenearifolia)*	0.002(0.001)	0.003(0.003)	1.55	Asteraceae
25	*Oenothera primiveris*	0.002(0.002)	0.003(0.002)	2.32	Onagraceae
26	*Astragalus allochrous*	0.001(0.0003)	0.001(0.0004)	4.7	Fabaceae
27	*Lappula occidentalis (=redowskii)*	0.001(0.0004)	0.005(0.003)	1.73	Boraginaceae
28	*Lesquerella (=Physaria) gordonii*	0.001(0.0002)	0.030(0.009)	0.77	Brassicaceae
29	*Ambrosia artemisiifolia*	0.0004(0.0003)	0.0005(0.0004)	3.99	Asteraceae
30	*Eschscholzia californica (=mexicana)*	0.0004(0.0002)	0.005(0.002)	1.5	Papaveraceae
31	*Nama hispidum*	0.0002(0.0001)	0.0001(0.0001)	NA	Boraginaceae
32	*Plagiobothrys arizonicus*	0.0002(0.0002)	0.002(0.001)	1.83	Boraginaceae
33	*Erodium texanum*	0.0002(0.0001)	0.010(0.009)	2.51	Geraniaceae
34	*Descurainia obtusa*	0.0001(0.0001)	0.00003(0.00003)	NA††	Brassicaceae
35	*Pectocarya recurvata*	0.00002(0.00002)	0.013(0.006)	0.95	Boraginaceae
36	*Nuttallanthus texanus (=Linaria taxana)*	0.00001(0.00001)	0.0001(0.00006)	0.02	Plantaginaceae

†Exotic species ††Not Available

Table S2: Winter annual plant species, mean (s.e.m.) fractional abundances observed in both pre-invasion and post-invasion time periods ranked from greatest to least abundant in the rodent removal plots, seed mass, and family.

Pre-invasion rank abundance	Species	Pre-invasion fractional abundance	Post-invasion fractional abundance	Seed mass (mg)	Family
1	*Machaeranthera (=Haplopappus) gracilis*	0.448(0.137)	0.004(0.002)	0.18	Asteraceae
2	*Astragalus nuttallianus*	0.134(0.040)	0.100(0.057)	1.53	Fabaceae
3	*Eriastrum diffusum*	0.091(0.023)	0.004(0.002)	0.19	Polemoniaceae
4	*Eriogonum abertianum*	0.055(0.016)	0.077(0.033)	0.22	Polygonaceae
5	*Erodium cicutarium*†	0.045(0.029)	0.687(0.091)	0.99	Geraniaceae
6	*Vulpia octoflora*	0.044(0.022)	0.011(0.005)	0.38	Poaceae
7	*Plantago patagonica (=purshii)*	0.030(0.014)	0.009(0.005)	1.19	Plantaginaceae
8	*Chaenactis stevioides*	0.027(0.016)	0.039(0.016)	0.47	Asteraceae
9	*Cryptantha micrantha*	0.017(0.010)	0.001(0.001)	0.02	Boraginaceae
10	*Spermolepis echinata*	0.015(0.008)	0.001(0.001)	0.62	Apiaceae
11	*Descurainia pinnata*	0.010(0.004)	0.007(0.003)	0.1	Brassicaceae
12	*Microseris (=Uropappus) lindleyi (=lenearifolia)*	0.010(0.008)	0.001(0.001)	1.55	Asteraceae
13	*Baileya multiradiata*	0.009(0.005)	0.001(0.001)	0.43	Asteraceae
14	*Lesquerella (=Physaria) gordonii*	0.008(0.003)	0.036(0.012)	0.77	Brassicaceae
15	*Lupinus concinnus*	0.006(0.003)	0.001(0.0004)	7.5	Fabaceae
16	*Gilia sinuata*	0.006(0.005)	0.0003(0.0003)	0.31	Polemoniaceae
17	*Phacelia arizonica (=popei)*	0.006(0.003)	0.0004(0.0003)	0.71	Boraginaceae
18	*Stephanomeria exigua*	0.005(0.002)	0.0004(0.0003)	0.64	Asteraceae
19	*Calycoseris wrightii*	0.005(0.003)	0.0001(0.0001)	0.72	Asteraceae
20	*Malacothrix fendleri*	0.004(0.003)	0.0001(0.0001)	0.1	Asteraceae
21	*Lepidium lasiocarpum*	0.004(0.002)	0.0004(0.0003)	0.3	Brassicaceae
22	*Chenopodium fremontii*	0.003(0.002)	0.002(0.002)	0.39	Chenopodiaceae
23	*Erodium texanum*	0.003(0.002)	0.006(0.001)	2.51	Geraniaceae
24	*Erigeron divergens*	0.002(0.001)	0.0003(0.0002)	0.03	Asteraceae
25	*Oenothera primiveris*	0.002(0.001)	0.002(0.001)	2.32	Onagraceae
26	*Eschscholzia californica (=mexicana)*	0.001(0.001)	0.001(0.0005)	1.5	Papaveraceae
27	*Cryptantha crassisepala*	0.001(0.0004)	0.001(0.001)	0.3	Boraginaceae
28	*Astragalus allochrous*	0.001(0.0006)	0.001(0.001)	4.7	Fabaceae
29	*Pectocarya recurvata*	0.0005(0.005)	0.001(0.0005)	0.95	Boraginaceae
30	*Lappula occidentalis (=redowskii)*	0.0002(0.0001)	0.0003(0.0003)	1.73	Boraginaceae
31	*Lupinus sp. 2*	0.0001(0.0001)	0.0001(0.0001)	NA††	Fabaceae
32	*Plagiobothrys arizonicus*	0.00002(0.0002)	0.0001(0.0001)	1.83	Boraginaceae

†Exotic species ††Not Available

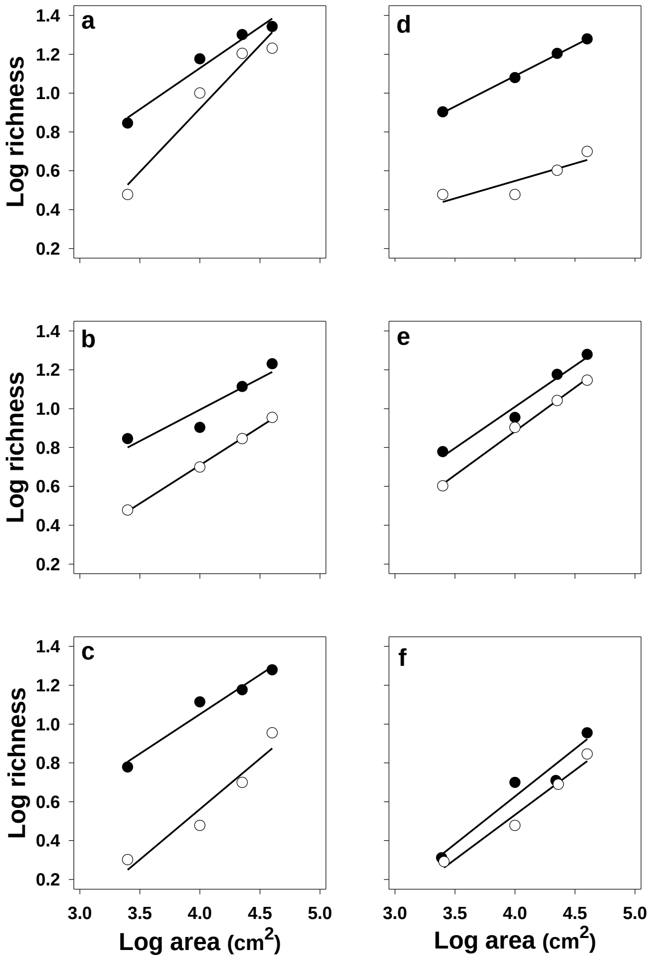

Figure S1: Species-area relationships for (a-c) three control and (d-f) three rodent removal plots. In each panel, one representative pre-invasion year (solid circles) and one post-invasion year (open circles) are depicted to illustrate how SAR slope and intercepts changed after invasion on the plot. Note that slopes tend to increase on control plots after invasion while slopes tend to be similar or lower on rodent removal plots after invasion. Also note that overall richness values are lower after invasion. Specific plot number, SAR slope and year are as follows: a) Plot 11: Pre-invasion slope (1995) = 0.42, post-invasion slope (2005) = 0.65; b) Plot 12: Pre-invasion slope (1994) = 0.32, post-invasion slope (2004) = 0.40; c) Plot 14: Pre-invasion slope (1993) = 0.41, post-invasion slope (2003) = 0.52; d) Plot 10: Pre-invasion slope (1993) = 0.31, post-invasion slope (2003) = 0.18; e) Plot 23: Pre-invasion slope (1995) = 0.42, post-invasion slope (2005) = 0.45; f) Plot 24: Pre-invasion slope (1994) = 0.50, post-invasion slope (2004) = 0.45.

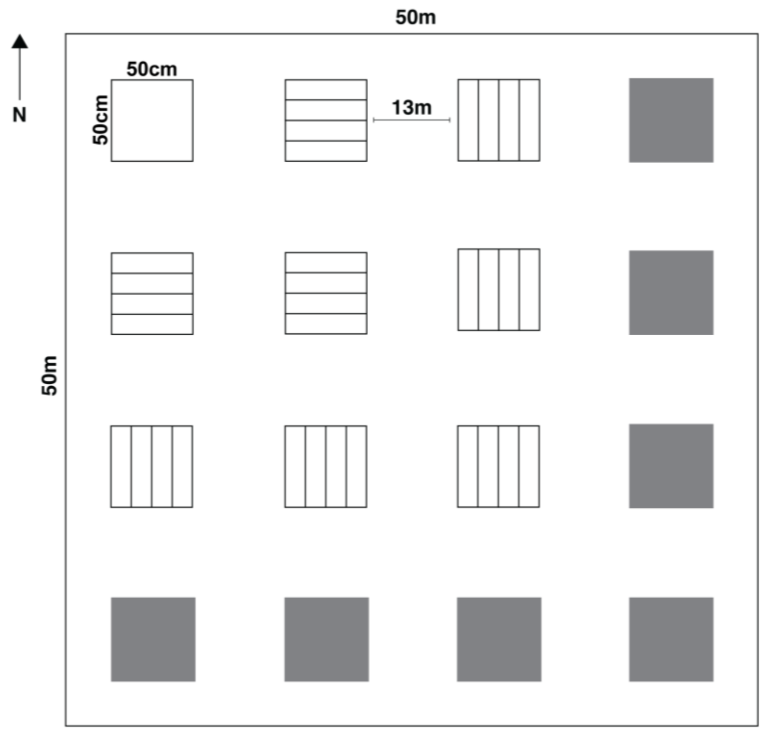

Figure S2: Species-area relationships were calculated at four spatial scales within each plot by combining adjacent quadrats. The smallest spatial scale used a single quadrat (open square, 25,000 cm2). The next largest scale added the three closest quadrats (horizontal bars, total area = 10,000 cm2). The next largest scale added the next five closest quadrats (vertical bars, total area = 22,500 cm2). The largest scale included the remaining seven gray squares, all 16 quadrats in the plot, equal a total area of 40,000 cm2.
